# A New Styptic

**Published:** 1867-12

**Authors:** 


A New Styptic.—An exchange says that the perchloride of iron combined with collodion is a good hæmostatic for wounds, the bite of leeches, etc. To prepare it, one part of crystallized perchloride of iron is mixed with six parts of collodion. The perchloride of iron should be added gradually and carefully, to prevent the evolution of excessive heat, which injures the collodion. The composition, when well made, is of a yellowish red color, perfectly limpid, and produces on the skin a yellow pellicle which retains great elasticity.

